# Supplementary material for: Characterization of the finch embryo supports evolutionary conservation of the naive stage of development in amniotes
Source: eLife. 2015 Sep 11;4:e07178. doi: 10.7554/eLife.07178 (PMC4608004; doi:10.7554/eLife.07178)
Supplement: Supplementary file 1. — (A) Details of primers that were used for generating in situ hybridization riboprobes for the zebra finch. (B) Details of primers that were used for quantitative PCR. DOI: http://dx.doi.org/10.7554/eLife.07178.010 [file elife07178s001.docx]

**SUPPLEMENTARY FILE 1**

**Supplementary File 1A**

**List of Primers used for generating *in situ* hybridisation riboprobes**

| **Gene Name** | **Targeted location** | **Primer Sequence** | | **Probe size (bp)** |
| --- | --- | --- | --- | --- |
| fDNMT3b | XM_002196272.1; 562-1078 | CAGGATGGGAAGGAATTTGGAA | GCTGCTCCTTGCTGCTGTTG | 517 |
| fGATA4 | XM_002186806.1; 249-925 | CCCTCCCGTGTCACCTCGGT | TCTCGCTGCTGGATGGGCCT | 677 |
| fGATA6 | XM_002194963.2; 379-952 | ATGTACCAGACCCTCGCCATC | TCTGGATGGAGCCGCAGTTG | 574 |
| fHHEX | XM_002189948.2; 1047-1798 | TTGCACTTTACCAGGGGCTGGTTT | ACGTTCTGCAGCTGATGCCAGTTT | 752 |
| fNANOG | XM_002190696.2; 31-984 | ATGAGCAGCCACCTAGGCAC | TCAGACCTGAGACTCATACCC | 954 |
| fNANOG-LIKE | XM_002190730.2; 135-894 | GGGGAGCAGGCACACAGCAGAAGTA | TTGGCACAGCCCCCACCTTCTCTT | 760 |
| fPOUV | XM_002190325.1; 65-689 | AGGCTGCGTGGCAGAAAGGTG | TAGGGCAGCAGCAGCCGTTTG | 625 |
| fSOX3 | XM_002199626.2; 913-1516 | TTACACAGCGTGCACCAGCA | CCCACCTCCCCCTTTTCAAG | 604 |
| fSOX17 | XM_004175276.1; 497-1465 | AGGACGAGCGCAAGCGGCT | CGCTGCTGGCGTCCGACAC | 969 |

**Supplementary File 1B**

**List of Primers used for Q-PCR**

| Gene Name  (f) finch,  (c) chick | Gene ID | Primer Sequences | | Size |
| --- | --- | --- | --- | --- |
| ZEBRAFINCH GENES | | | | |
| fCRIPTO | XM_002191122.1 | TTCACTGCGACTCTGGTCTG | TGCCGCTTTTGTGCTGTAAC | 107 |
| fDAZL | XM_002193965.2 | TCAAATTCCTGCGTGCAACC | CTGCTGCACGTATGTCTCCA | 102 |
| fDDX4 | XM_002186986.2 | ATCGAATTGGGCGAACTGGT | AGGTTGTGCAAGATGACCGT | 89 |
| fDNMT3B | [XM_002196272.1](http://www.ncbi.nlm.nih.gov/entrez/viewer.fcgi?db=nucleotide&id=224078172) | AGCCAAGAGGAGGCCAATTC | CACTTGGATGCCCAAGTCCT | 88 |
| fESRRB | [XM_002199964.2](http://www.ncbi.nlm.nih.gov/entrez/viewer.fcgi?db=nucleotide&id=449502652) | TCTCCAACCTGTCCCTTGGA | GCTTGTCCTCGTATGGCAGA | 105 |
| fFBXO15 | [XM_002192143.2](http://www.ncbi.nlm.nih.gov/entrez/viewer.fcgi?db=nucleotide&id=449493944) | GGATGCTGTGTCCCTGTTGA | TTGGGTGCAAAGAGCTGGAA | 107 |
| fFGF3 | [XM_002194521.1](http://www.ncbi.nlm.nih.gov/entrez/viewer.fcgi?db=nucleotide&id=224050479) | GCTCTACTGTGCCACCAAGT | CTGTTCTTCTCCAGGGTGCC | 75 |
| fFGF4 | [XM_004186226.1](http://www.ncbi.nlm.nih.gov/entrez/viewer.fcgi?db=nucleotide&id=449503864) | GTGCCTTTTTGCCACGTCTT | GGGCTACGCCTGTATGTGAA | 102 |
| fFGF5 | [XM_002191818.2](http://www.ncbi.nlm.nih.gov/entrez/viewer.fcgi?db=nucleotide&id=449499478) | ATTTGATGGCCTTTCGCAGC | GGAGAAGCCTTGGGGCTTAG | 106 |
| fFGF8 | [XM_002195841.1](http://www.ncbi.nlm.nih.gov/entrez/viewer.fcgi?db=nucleotide&id=224052699) | AATCAATGCGATGGCAGAGGA | GGCTCCCAAAGGTGTCTGTC | 71 |
| fFGF10 | [XM_002196864.2](http://www.ncbi.nlm.nih.gov/entrez/viewer.fcgi?db=nucleotide&id=449514179) | CCGTCTCTCCACTTGCCTTT | TGAAACTCTGGGCGTTGGAA | 84 |
| fFGF13 | [XM_004175772.1](http://www.ncbi.nlm.nih.gov/entrez/viewer.fcgi?db=nucleotide&id=449498364) | GCCAGCCAAGTGCTCTCTAA | CAACGGGAGGTTGGGATGAA | 76 |
| fGAPDH | [NM_001198610.1](http://www.ncbi.nlm.nih.gov/entrez/viewer.fcgi?db=nucleotide&id=310750351) | CCACATGGCATCCAAGGAGT | AGAGCTAAGCGGTGGTGAAC | 109 |
| fIL6 | [XM_002191284.2](http://www.ncbi.nlm.nih.gov/entrez/viewer.fcgi?db=nucleotide&id=449493049) | GGGCAAATGTGCGAGAAGTT | GTCACCTTGGGGAGGTTGAG | 83 |
| fKLF2 | [XM_002194040.2](http://www.ncbi.nlm.nih.gov/entrez/viewer.fcgi?db=nucleotide&id=449491706) | TATGCTGGCTGTGGGAAGAC | CAGTTGCAGTGGTAGGGCTT | 92 |
| fLIF | [XM_002186943.2](http://www.ncbi.nlm.nih.gov/entrez/viewer.fcgi?db=nucleotide&id=449477545) | TCCACAACACCACCAAGACC | TCCGTACCTGACATCCACCT | 98 |
| fLIN28A | [XM_002192235.1](http://www.ncbi.nlm.nih.gov/entrez/viewer.fcgi?db=nucleotide&id=224081662) | AAAGGTCTGGAGTCCATCCG | CTGGAGGCTCTTCCCTTTGG | 87 |
| fLIN28B | [XM_002196376.2](http://www.ncbi.nlm.nih.gov/entrez/viewer.fcgi?db=nucleotide&id=449497831) | GGGCGAGTAAAGGTGGAGAC | TGAACCATTTGCAATGGCCG | 99 |
| fMYC | [XM_004174202.1](http://www.ncbi.nlm.nih.gov/entrez/viewer.fcgi?db=nucleotide&id=449495156) | CAAGAGGCGAACACACAACG | CGTCACGCAAGGCAAAGAAA | 77 |
| fNANOG | [XM_002190696.2](http://www.ncbi.nlm.nih.gov/entrez/viewer.fcgi?db=nucleotide&id=449485126) | CCAGAGCACCTCTGACACTG | ATGGGGTGCCAAGGTAACTG | 78 |
| fNANOG-LIKE | [XM_002190730.2](http://www.ncbi.nlm.nih.gov/entrez/viewer.fcgi?db=nucleotide&id=449485130) | GGTTCCAGAACCGGAGGATG | GTAGGTACCGCTCTGGAAGC | 104 |
| fNR0B1 | [XM_002191941.2](http://www.ncbi.nlm.nih.gov/entrez/viewer.fcgi?db=nucleotide&id=449483057) | GAGTACGCTTACCTCAAGGGG | GCTTCCCTCTGCAGTCCTTC | 92 |
| fPOUV | [XM_002190325.1](http://www.ncbi.nlm.nih.gov/entrez/viewer.fcgi?db=nucleotide&id=224072936) | CTTCCGCAAGTGTGTGAAGC | AGAACCAGACCCGGACTACA | 92 |
| fPRDM14 | [XM_002197690.2](http://www.ncbi.nlm.nih.gov/entrez/viewer.fcgi?db=nucleotide&id=449494890) | CTCCACTGGCAACTGGATGT | AGATTTGTCCCTGGCACTGG | 89 |
| fSALL4 | [XM_002186584.2](http://www.ncbi.nlm.nih.gov/entrez/viewer.fcgi?db=nucleotide&id=449486484) | TAAAACCCACTACGGCGTCC | TTCTTCTGGCAAATGGGGCA | 75 |
| fSOX3 | [XM_002199626.2](http://www.ncbi.nlm.nih.gov/entrez/viewer.fcgi?db=nucleotide&id=449498777) | AATACTCGCTGCCCGGTAAC | AGCCGTTCATGTGGGCATAA | 105 |
| fTBX3 | [XM_004176567.1](http://www.ncbi.nlm.nih.gov/entrez/viewer.fcgi?db=nucleotide&id=449477019) | TCCAGCAGTAGGAAGACCGA | CCATGCTTGTCCCAGGGATT | 78 |
| fTFCP2L1 | [XM_002190309.1](http://www.ncbi.nlm.nih.gov/entrez/viewer.fcgi?db=nucleotide&id=224054679) | CCAAGAGAGGCACCTGGATG | TGCAGAGTCTGTGCCATGAG | 109 |
| CHICK GENES | | | | |
| cCRIPTO | NM_204700.2 | ACACACAAGACGGCTGTGAA | GGTGAGAAGGCAAATCAGCG | 107 |
| cDAZL | NM_204218.1 | TGTTTTTAAGTGTGCGGGCG | GCAGGTTGTTGACGAATGGG | 110 |
| cDDX4 | [NM_204708.2](http://www.ncbi.nlm.nih.gov/nucleotide/743405596?report=genbank&log$=nuclalign&blast_rank=1&RID=CH868DJ101R) | GGAGCCTGCAGTGATGTTCA | CGTTCACCACCTGTGCTTTG | 98 |
| cDNMT3B | [NM_001024828.1](http://www.ncbi.nlm.nih.gov/entrez/viewer.fcgi?db=nucleotide&id=67514586) | CAAACCACTGGGCGTCAAAG | ATGTTCAAGGCACAGCACCT | 94 |
| cESRRB | [XM_001235146.2](http://www.ncbi.nlm.nih.gov/entrez/viewer.fcgi?db=nucleotide&id=513188405) | CCTCATCCTGGGCATTGTGT | CCCGTCAGACGAGAGTGTTC | 99 |
| cFBXO15 | [XM_004935205.1](http://www.ncbi.nlm.nih.gov/entrez/viewer.fcgi?db=nucleotide&id=513171515) | GCTGTGTTCCTTTACCCCGA | TAGATCCAGCACCAACGCTC | 89 |
| cFGF3 | [NM_205327.1](http://www.ncbi.nlm.nih.gov/entrez/viewer.fcgi?db=nucleotide&id=46048689) | GATCAACGGCACCTTGGAGA | CCCTTGATAGCGACGATCCC | 84 |
| cFGF4 | [NM_001031546.1](http://www.ncbi.nlm.nih.gov/entrez/viewer.fcgi?db=nucleotide&id=71895928) | GGCGTCTCTATTGCAACGTG | TGTATCGGTTCTCGCTGTGG | 90 |
| cFGF5 | [XM_001233790.3](http://www.ncbi.nlm.nih.gov/entrez/viewer.fcgi?db=nucleotide&id=513183091) | GGGGATCGTAGGAATCCGAG | GCACTCGCATGGAGTTTTCC | 78 |
| cFGF8 | [NM_001012767.1](http://www.ncbi.nlm.nih.gov/nucleotide/61098219?report=genbank&log$=nuclalign&blast_rank=3&RID=8Y7ZMPKR013) | CAGCGGGAAGCACGTGCAGA | CACGCGGCTCCCAAAGGTGT | 112 |
| cFGF10 | [NM_204696.1](http://www.ncbi.nlm.nih.gov/entrez/viewer.fcgi?db=nucleotide&id=45382694) | GTGCGGAGCTACAATCACCT | TGACCTTGCCGTTCTTCTCG | 97 |
| cFGF13 | [NM_001001743.1](http://www.ncbi.nlm.nih.gov/entrez/viewer.fcgi?db=nucleotide&id=48976044) | TTCTGCCTTTTCGGCGTACA | CTGGACCCAGAGAAACCTCG | 77 |
| cGAPDH | [NM_204305.1](http://www.ncbi.nlm.nih.gov/entrez/viewer.fcgi?db=nucleotide&id=46048960) | CATCCAAGGAGTGAGCCAGG | CAGAACTGAGCGGTGGTGAA | 95 |
| cIL6 | [NM_204628.1](http://www.ncbi.nlm.nih.gov/entrez/viewer.fcgi?db=nucleotide&id=45382888) | AACAACCTCAACCTGCCCAA | GGAGAGCTTCGTCAGGCATT | 87 |
| cKLF2 | [XM_418264.4](http://www.ncbi.nlm.nih.gov/entrez/viewer.fcgi?db=nucleotide&id=513227814) | CCTTCCAGCAGAGCTACCAC | GTCCTCGAAAAGGCCGTACT | 95 |
| cLIF | [XM_425293.4](http://www.ncbi.nlm.nih.gov/entrez/viewer.fcgi?db=nucleotide&id=513211183) | CTTGCACCCGTCTCACAGAT | GCAGCATCAGCAGGAGGTAA | 88 |
| cLIN28A | [NM_001031774.2](http://www.ncbi.nlm.nih.gov/entrez/viewer.fcgi?db=nucleotide&id=82654185) | GGAGATTCACCCAAAGCCGA | TGCGGACGTTGAACCACTTA | 76 |
| cLIN28B | [NM_001034818.1](http://www.ncbi.nlm.nih.gov/entrez/viewer.fcgi?db=nucleotide&id=77736622) | TGGGAGCCCCTGTTTAGGAA | GGCCACCACAGTTGTAGCAT | 95 |
| cMYC | [NM_001030952.1](http://www.ncbi.nlm.nih.gov/entrez/viewer.fcgi?db=nucleotide&id=73661205) | GAGGAGAACGACAAGAGGCG | CACGCAGGGCAAAGAAACTC | 85 |
| cNANOG | [NM_001146142.1](http://www.ncbi.nlm.nih.gov/entrez/viewer.fcgi?db=nucleotide&id=225784818) | ACAGCTTGCAGGCAGAAGAT | CAAGGGGCCTGGTACTGATG | 100 |
| cNANOG-LIKE | ENSGALT00000045061 | TGCCTACCCCTTGTCAACAC | TCCTCCATCATTGCTGGTCAC | 71 |
| cNR0B1 | [NM_204593.1](http://www.ncbi.nlm.nih.gov/entrez/viewer.fcgi?db=nucleotide&id=46048922) | GTACGCCTACCTCAAGGGGA | TGTGCTTCCTTCTGCAGTCC | 93 |
| cPouV | [NM_001110178.1](http://www.ncbi.nlm.nih.gov/entrez/viewer.fcgi?db=nucleotide&id=158819066) | GAGGCAGAGAACACGGACAA | TTCCCTTCACGTTGGTCTCG | 109 |
| cPRDM14 | [XM_003640840.2](http://www.ncbi.nlm.nih.gov/entrez/viewer.fcgi?db=nucleotide&id=513172768) | GTTCGCCTACCGCTACTACC | CAGAGGTGGCAGGGGAATTT | 87 |
| cSALL4 | [NM_001080872.1](http://www.ncbi.nlm.nih.gov/entrez/viewer.fcgi?db=nucleotide&id=124249433) | TGACTCCTGGGCTGAGCTAT | TAGTGCCACGTATCGTCTGC | 78 |
| cSOX3 | [NM_204195.1](http://www.ncbi.nlm.nih.gov/nucleotide/45383725?report=genbank&log$=nuclalign&blast_rank=1&RID=CH7PVBNF01R) | GAAGGACAAATACTCGCTGCC | TGGGCGTAAGTGTCAATCCTC | 102 |
| cTBX3 | [NM_001270878.1](http://www.ncbi.nlm.nih.gov/entrez/viewer.fcgi?db=nucleotide&id=399567822) | GTGGAAGACGACCCGAAAGT | CACCATCTCCGTGCCTCTTT | 78 |
| cTFCP2L1 | [XM_422087.4](http://www.ncbi.nlm.nih.gov/entrez/viewer.fcgi?db=nucleotide&id=513194906) | TCAGCACATTAAAAGCTGAAAGCA | AGCAATCTCAGTGAGGCACTA | 110 |
